# Mesenchymal stem cells-based therapies for severe ARDS with ECMO: a review

**DOI:** 10.1186/s40635-024-00596-w

**Published:** 2024-02-09

**Authors:** Jing-Ke Cao, Xiao-Yang Hong, Zhi-Chun Feng, Qiu-Ping Li

**Affiliations:** 1 Department of Neonatology, Senior Department of Pediatrics, the Seventh Medical Center of PLA General Hospital, NO. 5 Nanmencang, Dongcheng District, Beijing, 100700 China; 2 The Second School of Clinical Medicine, Southern Medical University, Guangzhou, 510515 China; 3 Department of Pediatric Intensive Care Unit, Senior Department of Pediatrics, the Seventh Medical Center of PLA General Hospital, NO.5 Nanmencang, Dongcheng District, 100700 Beijing, China

**Keywords:** Respiratory distress syndrome (RDS), Extracorporeal membrane oxygenation (ECMO), Mesenchymal stem cells (MSCs), Coronavirus disease 2019 (COVID-19)

## Abstract

Acute respiratory distress syndrome (ARDS) is the primary cause of respiratory failure in critically ill patients. Despite remarkable therapeutic advances in recent years, ARDS remains a life-threatening clinical complication with high morbidity and mortality, especially during the global spread of the coronavirus disease 2019 (COVID-19) pandemic. Previous studies have demonstrated that mesenchymal stem cell (MSC)-based therapy is a potential alternative strategy for the treatment of refractory respiratory diseases including ARDS, while extracorporeal membrane oxygenation (ECMO) as the last resort treatment to sustain life can help improve the survival of ARDS patients. In recent years, several studies have explored the effects of ECMO combined with MSC-based therapies in the treatment of ARDS, and some of them have demonstrated that this combination can provide better therapeutic effects, while others have argued that some critical issues need to be solved before it can be applied to clinical practice. This review presents an overview of the current status, clinical challenges and future prospects of ECMO combined with MSCs in the treatment of ARDS.

## Main text

Acute respiratory distress syndrome (ARDS) is a devastating disease caused by a variety of intrapulmonary or extrapulmonary factors, such as infection, trauma and shock, resulting in diffuse injuries to alveolar epithelial cells and capillary endothelial cells. ARDS has high morbidity and mortality, and is the primary cause for poor prognosis in critically ill patients [1]. According to the LUNG SAFE study, ARDS accounts for 10.4% of all intensive care unit admissions, with a mortality rate ranging from 36.4 to 87% depending on the severity of the disease, and severe ARDS accounts for 23% of all cases with a mortality rate of 46.1% [2–4]. Those who survived face physical and mental sequelae, reduced quality of life and higher medical costs [5]. With the progress of the times, the understanding of ARDS has become more profound. Whether it is early wet lung, shock lung, or later adult respiratory distress syndrome, people have gradually come to realize that ARDS is no longer a simple lung disease, but a mixture of multiple diseases, and an important part of multiple organ dysfunction syndrome also [6]. Because of this, it is difficult to define the focus of therapeutic strategies. Currently, the main treatments for ARDS mainly include diagnosis and treatment of infections, protective mechanical ventilation strategies, prone positioning, sedation management, fluid therapy, and extracorporeal membrane oxygenation (ECMO) [1]. However, these are mostly some supportive measures, and no targeted drug can effectively improve the clinical outcome, especially for severe and critically ill ARDS patients. Due to the coronavirus disease 2019 (COVID-19) pandemic, the global incidence of ARDS is likely to increase further, and about 67–85% critically ill patients infected with COVID-19 have developed ARDS with a mortality rate of over 60% [7, 8]. Therefore, it is imminent to search for effective treatment strategies.

## Application of ECMO in ARDS

Since the influenza A (H1N1) pandemic in 2009, the H7N9 avian influenza epidemic in 2013, and especially the global outbreak of COVID-19 infection in 2020, ECMO has been increasingly used as a rescue and supportive treatment for ARDS [9, 10]. As ECMO can improve oxygenation and removal of carbon dioxide, reduce ventilator support, and allow the lungs to rest, thereby reducing ventilation-induced lung injury, it is regarded as a life-saving treatment for critically ill patients and some studies have shown that ECMO can reduce the 60-day mortality rate of critically ill ARDS patients [11, 12]. However, the benefit of ECMO in severe ARDS patients has long been debated. The EOLIA trial, a large randomized controlled study evaluating the efficacy of early ECMO in the treatment of ARDS [13], showed a slight reduction in 60-day mortality with ECMO (35% vs. 46%, *P* = 0.09). This may be because 28% patients in the non-ECMO treatment group finally used ECMO due to severe hypoxemia during the experiment, making the effect of the ECMO treatment group not significant [13]. Subsequent Bayesian and meta-analyses of this trial suggested that early ECMO use helped reduce mortality in patients with severe ARDS [14, 15]. The therapeutic efficacy of ECMO may be influenced by many factors, such as the etiology and severity of ARDS, whether adjuvant therapy such as prone position ventilation is used, and the setting of ventilator parameters during use [12, 16, 17]. In addition, age, gender and body mass index are also thought to be associated with mortality [16]. For critically ill patients, prolonged use of ECMO support increases the occurrence of complications such as bleeding, thrombosis, organ failure, and infection [18]. A follow-up survey reported that 36% patients still had persistent dyspnea, and 30% patients continued to take pulmonary drugs after discharge [19]. Another explanation for the current high mortality rate with ECMO is the complex immune damage induced during ECMO bypass [19]. When the ECMO device is placed in the body it will activate various coagulation and inflammatory responses in the body, resulting in a rapid increase in the level of pro-inflammatory factors, causing dysfunction of multiple organ systems in severe cases [20]. Despite significant improvements in ECMO devices (pump, cannula design and oxygenator) and heparin-coated tubing, inflammatory responses are unavoidable. Some studies [21, 22] reported that inflammatory factor levels in severe ARDS patients were higher than those in mild, indicating that controlling the inflammatory storm during ECMO in ARDS patients may help improve the condition.

## Mesenchymal stem cells (MSCs) therapy in ARDS patients

Intensive efforts have long been made to develop effective drugs for the treatment of ARDS. In recent years, MSCs have received increasing attention as a means of treating ARDS. MSCs are multipotent adult stem cells which can be isolated from various tissues and organs, and the most common sources for the treatment of respiratory diseases are bone marrow (BM), umbilical cord blood (UCB), adipose tissues (AT) and endothelial progenitor cells [23]. MSCs have the advantages of a self-renewal ability, low immunogenicity, and an ability to home to damaged tissues [24]. The role of MSCs mainly depends on their paracrine mechanism, which can produce a variety of immunomodulatory soluble factors to regulate inflammatory responses and reduce lung injury [25]. In addition, it can not only increase pulmonary fluid clearance by secreting keratinocyte growth factor, but also promote the regeneration of type II alveolar epithelial cells by secreting large amounts of angiopoietin-1 and hepatocyte growth factor, thereby restoring the epithelium cell barrier function [26–28]. Another major mechanism is associated with their homing ability. Chemokines such as stromal cell-derived factor-1 released from the injured lung tissue could mediate targeted migration of MSCs to the injured lung tissue by binding to ligands such as CXC-chemokine receptor type-4 released by MSCs [29].

The safety of MSCs in the treatment of ARDS has been validated in many clinical studies. Zheng et al. recruited 12 patients with moderate-to-severe ARDS and randomly divided them into a treatment group receiving a single intravenous infusion of 1 × 10 ^6^cells/kg of AT-derived MSCs (AT-MSCs), and a control group receiving the same amount of normal saline within 48 h after enrollment [30]. This study provided the first demonstration that MSCs are safe and well tolerated in ARDS patients. In a later double-blind randomized controlled trial of COVID-19 ARDS, subjects received two intravenous infusions of (100 ± 20) × 10^6^ cells UCB-derived MSCs (UCB-MSCs) and no serious adverse events related to UCB-MSCs infusion were observed [31]. Other studies have also had encouraging results [32, 33]. Few clinical studies have focused on the assessment of therapeutic effect of MSCs in ARDS. Dilogo et al. enrolled 20 ARDS patients infected with COVID-19, and gave intravenous infusion of 1 × 10^6^cells/kg UCB-MSCs and found that the survival rate of the UCB-MSCs group was 2.5 times that of the control group [34]. They also found that UCB-MSCs transfusion could significantly reduce the level of inflammatory factor IL-6 in the patients. In another ARDS study for COVID-19, patients received 2 high-dose UCB-MSCs infusions of (100 ± 20) × 10^6^ cells/kg each time, and the finding was that UCB-MSCs could effectively reduce mortality and shorten the recovery time [31]. Other studies could not provide reliable assessment on the efficacy of treatment because of some research limitations [30, 35]. To further evaluate the efficacy of MSC treatment, Willson et al. conducted a 2b trial (NCT03818854) in a larger number of patients. A recent meta-analysis also concluded that MSCs can reduce mortality in patients with ARDS [36]. Despite the heterogeneity of these clinical trials, MSCs have generally shown a certain potential in the treatment of ARDS. However, few studies used MSCs to treat the most severe ARDS patients who required ECMO. These patients are the ones who fail to respond to traditional treatments and have the highest mortality rates, and often need ECMO treatment the most.

## Current situation and prospect of ARDS treatment based on MSCs combined with ECMO

With the widely application of MSCs and the development of ECMO in the treatment of ARDS, we believe that combined use of the two can maximize the benefits in that ECMO and MSCs can interact mechanistically. The increased level of inflammatory factors and the decreased number and function of lymphocytes during ECMO may be the factors affecting the mortality of ARDS patients [19], while MSCs can play an immunomodulatory function by inhibiting the Wnt/β-catenin pathway to inhibit the apoptosis of resident cells and immune cells, thus serving as a potential supportive therapy to alleviate the adverse reactions of ECMO [37], thus promoting disease recovery and reducing ECMO time. In a previous animal experiment, the authors found that fetal membrane hematopoietic stem cells could reduce the systemic inflammatory response in rat cardiopulmonary bypass (CPB) by inhibiting the expression of inflammatory cytokines and promoting the expression of protective factors in the lung [38]. Therefore, the anti-inflammatory effect of MSCs may be an effective method to control the inflammatory response during ECMO. ECMO has also been shown to mobilize MSCs in patients with ARDS [39]. Hoesli et al. isolated MSCs from the peripheral blood of term neonates requiring ECMO treatment [40], and Patry et al. subsequently found that the numbers of the MSC subpopulations CD34^−^/CD73^+^/CD90^+^ and CD34^−^/CD73^+^/CD29^+^/CD90^+^ in adult patients with ARDS treated with ECMO were significantly higher than that in non-ECMO treated patients [39]. Whether this mechanism is due to the relatively stable Ang2 level during ECMO promoting the regeneration of MSCs remains to be investigated [39].

In animal research, Kocyildirim et al. used *E. coli* to create a sheep model of ARDS and instilled multipotent adult progenitor cells in the trachea [41], followed by ECMO support and 6 h observation. They found that the anti-inflammatory factor IL-10 in the blood of sheep in ECMO plus multipotent adult progenitor cells group was increased, and the levels of inflammatory factors IL-6, IL-1β and IL-8 were decreased. (Table 1) In addition, the lung tissue sections also showed a blank compared with those in ECMO group and ARDS model, while neutrophil infiltration and hemorrhagic changes were significantly milder in the control group. In another sheep trial of MSCs combined with ECMO for ARDS [42], Millar et al. instilled MSCs and cell-free carriers into the bronchi after 1-h ECMO placement, and found that although MSCs reduced lung injury and inflammatory responses, the adhesion of MSCs to the external oxygenator fiber increased the transmembrane pressure and caused a rapid decline of the oxygenator performance. Therefore, it did not improve the oxygenation or ventilation parameters in the sheep model, the same as their previous ex vivo experiments. The fact that MSC adhesion affects the function of the oxygenator raises concerns and poses challenges to the combination therapy of MSCs and ECMO.

**Table 1:** Related clinical studies, case reports and animal studies of mesenchymal stem cells combined with extracorporeal membrane oxygenation for ARDS treatment

Reference	Type of study	Model/causes	Type of ECMO	MSC Sources	Patient/animal enrolled	Transplantation	Regimen	Main findings
Lin et al. [75]	Clinical study	Adenovirus pneumonia	VV-ECMO and VA-ECMO	BM-MSCs	1	i.t	6.25 × 10^6^cells/kg, single dose	Respiratory and lung condition improved
Kaushal et al. [45]	Clinical study	COVID-19	VV-ECMO	BM-MSCs	40(9/31)	i.v	2–3 doses	↓Mortality,COVID-19 IgG Spike protein, the SOFA score, VEGF-A, IL-12-p70 ↑PaO_2_/FiO_2_
Simonson et al. [43]	Clinical study	A H1N1	VV-ECMO; VA-ECMO	BM-MSCs	2	i.v	2 × 10^6^ cells/kg, Single dose	oxygenation and pulmonary compliance improved ↓ IL-6,IL-8,IFN-γ ↑Granulocyte macrophage colony-stimulating factor levels, surfactant protein B
Jungebluth et al. [76]	Clinical study	Burn	VV-ECMO and VA-ECMO	PBMCs and EPO	1	i.t	(300 ± 50) × 10^6^ PBMCs, on ECMO days 9, 10, 16, 21 and 23, total 5 dose and 30,000 IU EPO every other day, from ECMO day 9 to ECMO day 20, three times per day, total 18 doses	Further regression of inflammation in bronchoscopy images ↓WBC ↑CD14^+^,CD83^−^, miR-449a, b, c and miR-34a
Liu et al. [47]	Clinical study	*Pneumocystis carinii *pneumonia	VV-ECMO	UCB	1	i.t	nucleated cells:0.77 × 10^6^cells/kg live CD34 cells: 0.25 × 105cells/kg, single dose	Lung lesions and ECMO condition improved ↑CD16 + ,CD56 + ↓IL-α,IL-6,IL-8,IL-10,IL-1β, PCT
Tao et al. [74]	Clinical study	COVID-19	Unknown	UCB-MSCs	1	i.t	1.5 × 10^6^cells/kg, Every 48 h, total 5dose	PaO_2_/FiO_2_ ratio maintained stable ↓Blood creatinine, urea nitrogen ↑Pulmonary static compliance
Millar et al. [42]	Animal study	Border Leicester Cross ewes, oleic acid i.v. and *E. coli* LPS i.t	VV-ECMO	iPSC-derived hMSCs	14(7/7)	i.t	After 1 h of VV-ECMO, 3 × 10^8^iPSC-derived hMSCs, Single dose	the membrane oxygenator function is impaired ↓Lung inflammation, lymphocyte count,IL-8, the depth and severity of shock ↑Pulmonary arterial thromboses
Kocyildirim et al.^41^	Animal study	Sheep, *E. coli* endotoxin	VV-ECMO	MAPC	11(5/3/3)	i.t	After 1.5 h of the infusion of E. coli endotoxin, followed by VV-ECMO support; 40 million cells; Single dose	↑Pulmonary artery pressure, neutrophils counts Less inflammatory and hemorrhagic change in lungs

*ARDS* acute respiratory distress syndrome, *ECMO* extracorporeal membrane oxygenation, *MSC* mesenchymal stem cell, *BM-MSCs* bone marrow-derived mesenchymal stem cells, *i.t.* intratracheal, *i.v.* intravenous, *SOFA *Sequential Organ Failure Assessment, *VEGF *vascular endothelial growth factor, *IL *interleukin, *PBMCs* peripheral blood-derived mononuclear cells, *EPO* erythropoietin, *WBC* white blood cell, *UCB* umbilical cord blood, *PCT *procalcitonin, *COVID-19* coronavirus disease 2019, *UCB-MSCs* umbilical cord blood-derived mesenchymal stem cells, *iPSC* induced pluripotent stem cell, *hMSCs* human mesenchymal stem cells, *MAPC *multipotent adult progenitor cells

Position: at the end of “Current situation and prospect of ARDS treatment based on MSCs combined with ECMO” paragraph

At present, there are few clinical studies on the treatment of ARDS by MSC combined with ECMO, and most studies have used MSCs in the later stages of ECMO, despite they have exhibited positive effects. Simonson et al. treated two adult patients with refractory ARDS requiring VV-ECMO therapy with a single central vein infusion of allogeneic BM-derived MSCs(BM-MSCs) at a total dose of 2 × 10^6^ cells/kg, and both showed clinical improvement in lung function and were eventually discharged from the hospital [43]. They were physical and mental recovery during the 5-year follow-up after discharge, with no sign of pulmonary fibrosis [44]. Kaushal et al. [45] reported on 12 ARDS patients with COVID-19 infection treated with BM-MSCs, and none of the 9 patients who underwent VV-ECMO treatment experienced any MSC-associated adverse effects associated. Compared with the control group receiving VV-ECMO alone, their combination therapy not only more effectively reduced the overall mortality (22.2% vs 48.4%, *P* = 0.25) but improved the oxygenation index and inflammatory factor levels within a few days of MSC infusion. This recent clinical trial seems provide strong evidence for ECMO combination with MSCs therapy under these conditions. In addition, several studies have reported combination therapy of using UCB, knowing that it is biologically close to embryonic stem cells and has a higher proportion of hematopoietic progenitor cells and hematopoietic stem cells [46]. In addition, UCB has advantages of easy access, rapid use and no harm to the donor. Liu et al. [47] successfully treated a pediatric patient with ARDS with severe pulmonary sporotrichia pneumonia using UCB after 30-day failure of response to full-flow VV-ECMO supportive therapy and his lung function was significantly restored at subsequent follow-up. These examples shown that MSCs not only have good therapeutic efficacy in patients with severe ARDS but have better therapeutic effects in some patients with refractory ARDS who require ECMO treatment.

## Barriers and future directions for MSCs combined with ECMO in ARDS treatment

**Fig. 1:**
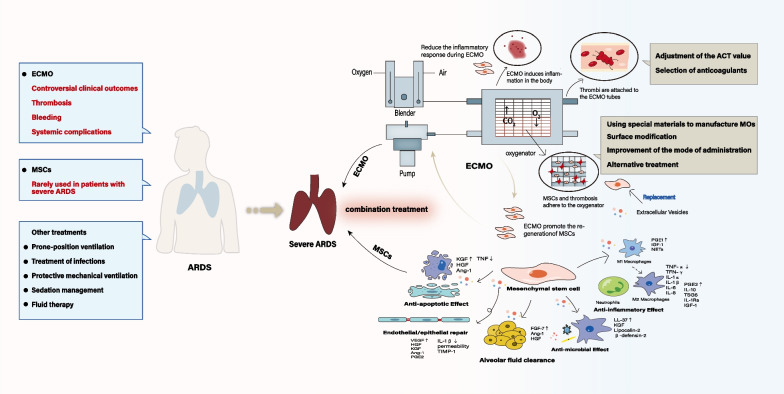
Mechanism, existing problems and solutions of ECMO combined with MSCs in the treatment of severe ARDS. Current treatment strategies for ARDS, and the interaction, problems and solutions of ECMO combined with MSCs in the treatment of severe ARDS. *ECMO* extracorporeal membrane oxygenation, *MSCs* mesenchymal stem cells, *ARDS* acute respiratory distress syndrome, *ACT* activated clotting time, *MOs* membrane oxygenators, *KGF* keratinocyte growth factor, *HGF* hepatocyte growth factor, *Ang-1* angiopoietin-1, *TNF* tumor necrosis factor, *VEGF* vascular endothelial growth factor, *PGE2* prostaglandin E2, *IL* interleukin, *TIMP-1* tissue inhibitor of metalloproteinases 1, *FGF-7* fibroblast growth factor 7, *PGE1* prostaglandin E1, *NETs* neutrophil extracellular traps, *TSG6* tumor necrosis factor-alphastimulated gene 6, *IGF-1 *Insulin-like growth factor 1, *LL-37* leucine leucine 37

Although some studies have brought hope to this new type of treatment, many urgent problems need to be solved in addition to evaluating the effect of treatment (Fig. 1). The first is the compatibility between MSCs and ECMO oxygenators. Several studies have raised the concern about MSC adhesion to the oxygenator surface, worrying that it may affect the performance of the oxygenator [42]. Millar et al. [48] simulated an ex vivo model of ECMO and intravascular administration of BM-MSCs, and found that the blood flow of the ECMO oxygenator decreased by 25% and the pressure gradient difference before and after use of the oxygenator increased correspondingly within 4 h after infusion. In addition, MSCs were found attached to the plastic fibers of the oxygenator. They believed that intravenous administration would increase the unnecessary systemic distribution of MSCs, so they used intratracheal administration in the subsequent study, but the same problem occurred [42]. In a later study [38], Takuya et al. simulated the CPB device used in pediatric cardiac surgery, and infused BM-MSCs into the CPB device, while electron microscopy did not find MSCs attached to the surface of the oxygenator and the cellular viability of MSCs was not affected as well. They believed this may be related to the different oxygenator materials and braid winding techniques used in the study. People have always been looking for ways to improve the biocompatibility of membrane oxygenators (MOs) with the human body, and using special materials to manufacture MOs is one of the methods. There are mainly three kinds of MO materials currently used: silica gel, polypropylene (PP) and polymethylpentene (PMP). Due to the poor performance of silicone MO, they have been gradually replaced by PP and PMP MO. Compared with PP, PMP has stronger tightness and its incidence of plasma leakage is smaller than that of PP, and therefore it is therefore not prone to failure and is suitable for long-term use. But Laluppa and colleagues have previously demonstrated that MSCs have strong adhesion to PMP [49]. Several studies explored the cause of MSC adhesion in PMP-based MO, but no specific mechanism was defined. Knowing that MSCs are a type of large cells with a diameter of 10 μm to 30 μm, Zhang et al. [50] proposed the use of MSC-derived extracellular vesicles (EVs) in ECMO instead of MSCs. EVs have a diameter of 30 nm-100 nm, which is much smaller than the diameter of MSCs and the pore size in MO could potentially avoid sticking to the oxygenator to impair the function of the oxygenator. Some animal models showed that EVs were as effective as MSCs in pulmonary vascularization and alveolarization [51, 52]. In addition, through the improvement of the drug delivery method, Liu et al. temporarily clamped the outflow channel of ECMO when infused UCB into the femoral vein, so that hematopoietic stem cells could flow to the pulmonary circulation as much as possible and avoid delivery to the ECMO pipeline [47]. Their experiences are worth learning from.

The second is thrombosis. When the human blood comes into contact with the artificial ECMO cannula, fibrinogen and albumin in the body will adhere to the cannula surface and activate platelets and thrombin, leading to thrombosis [53]. In adult patients receiving ECMO treatment, coagulation dysfunction may be as high as 33%, and MSC can activate coagulation pathways by expressing tissue factors (TF) and other mechanisms [54, 55]. So, anticoagulation management cannot be ignored in ECMO combination with MSCs therapy. To reduce the procoagulant activity of the coagulation system and prevent thrombosis during ECMO, exogenous systemic anticoagulation and heparin-coated MO and ECMO pipelines are usually employed. The most widely accepted "gold standard" is continuous intravenous micropump infusion of heparin for anticoagulation. Heparin has the advantages of a precise anticoagulant effect, a short half-life, antagonism by protamine and easy accessibility, but it also increases the risk of heparin-induced thrombocytopenia. The heparin surface coating has also reported to increased fibrin adhesion [49, 56]. More importantly, it may interfere with the homing and migration of MSCs to the lung tissue by blocking SDF-1-CXCR4 signaling pathway [57]. Thrombin inhibitors such as bivalirudin do not interfere with this signaling pathway, and have become a new trend in anticoagulation therapy in CPB in recent years, and may replace the anticoagulant function of heparin in combined therapy [57]. Stephenne and colleagues suggest that dual anticoagulants of heparin and bivalirudin can control the procoagulant activity of MSCs to improve therapeutic efficacy [58]. In addition to this, it is also crucial to develop reliable anticoagulation standards. The anticoagulation strategy proposed by the Extracorporeal Life Support Organization recommends a heparin bolus of 50–100 U/kg at intubation, followed by an infusion of 7.5–20 U/kg/h, and the target of whole blood activated clotting time is about 180–220 s or partial thromboplastin time adjusted within the target range of 1.5–2.5 times normal 60–80 s [59]. To prevent thrombosis, Takuya et al. maintained a high target activated clotting time value and controlled activated clotting time above 400 s in their in vitro experiment [38]. Other studies have suggested that elevated coagulation potential in some severe disease states may lead to "functional" heparin resistance [60]. Therefore, in patients with severe ARDS requiring ECMO treatment, higher doses of heparin may be required to reduce heparin resistance and thus reduce thrombosis during ECMO. In addition, the procoagulant activity shown by MSCs from different sources is also different. AT-MSCs appear to express more TF than BM-MSCs and have a stronger procoagulant effect [61]. Care should be taken to select cell sources that express less TF when used in clinical settings.

Finally, to improve the therapeutic efficacy of MSCs in ARDS patients using ECMO, several issues regarding MSCs need to be considered, including the timing and method of MSC administration, the source of MSCs, and the cost. Most current studies on combination therapy of MSCs and ECMO reported addition of MSCs when ECMO therapy was ineffective during the late hospitalization period, believing that it could produce significant short-term results. But as ARDS is an acute pathological process, whether administration of MSCs in the early stage of the disease could be more helpful in reducing ARDS inflammatory storm and adverse effects of ECMO itself on the body remains to be validated. The least fibrosis is observed in the exudative phase of ARDS onset, that is, MSCs therapy within 7 days of ARDS diagnosis may be appropriate [35]. In terms of the availability of MSCs, they can be obtained from different tissues and there are functional variations between tissues [62]. UCB-MSCs and BM-MSCs show better therapeutic potential than AT-MSCs in acute lung injury animal models [63]. Compared with adult tissues, MSCs cultured from neonatal tissues have a longer viable lifespan, a higher proliferation rate and higher differentiation potential [64]. Selecting sources of MSCs with specific biological properties may help improve the therapeutic efficacy. For example, severe acute respiratory syndrome coronavirus 2 is mainly caused by the combination of S protein on the surface of the virus and angiotensin-converting enzyme 2 on the surface of the cell to enter the host cell and cause disease, while UCB-MSCs, BM-MSCs and AT-MSCs do not express the angiotensin-converting enzyme 2 receptor and therefore can be used to treat severe ARDS induced by severe acute respiratory syndrome coronavirus 2 [65]. Intravenous is the most widely used route of administration because it is less invasive and can be repeated [66]. Most cells injected via the intravenous route are retained in the lung due to the first-pass effect, thereby prolonging their persistence in the lung, which is most beneficial for the treatment of lung diseases [67]. Although some studies reported the phenomenon of oxygenator adhesion in their vitro experiments, they may not have performed the gas exchange experiment of the oxygenator and ignored the effect of MSCs on the lung. Intratracheal administration can directly deliver MSCs to the damaged site, thus reducing the systemic distribution of MSCs, which may be theoretically more beneficial to ECMO patients. However, adhesion problems have also occurred in these animal studies [42], but the effects in current clinical applications are similar whether by intratracheal or intravenous administration, and no adhesion problem has been reported. The optimal dose of MSCs has not yet been determined. Most studies have confirmed that a single dose of 1 × 10^6^ cells/kg is safe, and the safe dose in other clinical studies ranges from 1 × 10^6^ cells/kg to 400 × 10^6^ cells/kg. Due to the short residence time of MSCs in the lung tissue after administration, very few MSCs play a role [25], so some studies chose multiple administrations to prolong the action time. Although a single dose has achieved good therapeutic effects, it may be possible to further improve lung repair function with multiple doses based on half-life [68]. Recent studies have shown that the cell activity of MSC can be improved by cryopreservation in combination with cell cycle [69]. In addition, the high-cost problem is also an obstacle for future MSC application. ECMO treatment itself is an advanced and expensive technique that is highly dependent on specialized personnel and advanced technology. According to statistics, ECMO hospitalization costs range from $22,305 to $334,608 [70]. However, the conditions for the preparation of MSCs are strict and the number of effective MSCs cells extracted from bone marrow or other sources is small each time, so it also increases the cost of treatment [71]. In recent years, the use of MSC-EVs and mononuclear cells to replace MSCs has been investigated. They are more accessible and easier to store [72]. In addition, they can be prepared in advance for rapid clinical use when needed and EVs also have a longer half-life than MSCs [73], and both MSCs and EVs have shown good therapeutic effects in clinical practice [47, 74].

## Conclusion

The high morbidity and mortality rates of ARDS and the lack of specific medications remain formidable clinical challenges at present. The number of patients with severe ARDS has risen since the outbreak of COVID-19, for whom safe more effective and affordable therapeutic strategies are urgently needed. The safety and efficacy of MSCs and ECMO in the treatment of ARDS have been investigated in phase 1 and 2 clinical trials, and some of them have ready advanced to phase 3 trials. ECMO combined with MSCs therapy for severe ARDS cases, especially those complicated by COVID-19 infection are still in the infant stage. Although some studies have demonstrated the therapeutic potential of combination therapy of MSCs and ECMO, more randomized controlled trials are required to confirm the safety and efficacy of combination MSCs and ECMO therapy. There are still numerous issues that need to be addressed to achieve the optimal therapeutic effect before it can be used in clinical practice.

*Take-home message:* This paper discusses the recent progress of ECMO combined with MSCs in the treatment of severe ARDS.

## Data Availability

Not applicable.
